# Panitumumab-Associated Encephalopathy after Accidental Intra-arterial Application through Dislocated Central Venous Access Device

**DOI:** 10.3389/fneur.2016.00196

**Published:** 2016-11-07

**Authors:** Slaven Pikija, Georg Pilz, Gerald Gschwandtner, Cornelia Rösler, Konstantin Schlick, Richard Greil, Johann Sellner

**Affiliations:** ^1^Department of Neurology, Christian Doppler Medical Center, Paracelsus Medical University Salzburg, Salzburg, Austria; ^2^Department of Geriatric Medicine, Christian Doppler Medical Center, Paracelsus Medical University Salzburg, Salzburg, Austria; ^3^Laboratory of Immunological Molecular Cancer Research, Department of Internal Medicine III with Hematology, Medical Oncology, Hemostaseology, Infectious Diseases, Rheumatology, Oncologic Center, Paracelsus Medical University Salzburg, Salzburg, Austria; ^4^Department of Neurology, Klinikum rechts der Isar, Technische Universität München, München, Germany

**Keywords:** panitumumab, immune-mediated, encephalopathy, accidental arterial infusion, cancer

## Abstract

Acute central nervous system (CNS) toxicity and immune-related side effects are increasingly recognized with the use of monoclonal antibodies for cancer therapy. Here, we report a patient who developed of acute-onset encephalopathy and coma, which began shortly after administration of panitumumab for the treatment of metastatic colorectal cancer. Echocardiography revealed that the drug had been infused into the left cardiac ventricle *via* a dislocated central venous line. Diffusion-weighted magnetic resonance imaging disclosed multiple cortical hyperintensities, which were preferentially located in the frontal lobes. While the neurological condition improved within a few days, the patient died 4 weeks later. It seems likely that the administration of the antibody *via* the intra-arterial route contributed to the development of this condition. Toxic encephalopathy may be a hitherto unrecognized complication of panitumumab treatment and should be taken into consideration in patients developing CNS symptoms undergoing this therapy.

## Introduction

Colorectal cancer is the second leading cause of cancer mortality in the developed world and third most common cancer worldwide (1). Epidermal growth factor receptor (EGFR) is a key therapeutic target, given that its activation stimulates key signaling processes involved in tumor growth and progression. Monoclonal antibodies against EGFR including cetuximab (Erbitux©, Merck, KGaA, Darmstadt, Germany) and panitumumab (Vectibix©, Amgen Inc., Thousand Oaks, CA, USA) have emerged to an important component in the treatment regimen for metastatic colorectal cancer. Considerable improvements in both progression-free survival and overall survival were achieved with the introduction of these therapies to 5-fluorouracil (5-FU)-based chemotherapy either in combination with oxaliplatin or irinotecan (2, 3). Panitumumab, a fully human monoclonal IgG2 antibody specific to the extracellular domain of EGFR, is approved for the treatment of EGFR-expressing metastatic colorectal cancer in patients with non-mutated (wild-type) V-Ki-ras2 Kirsten rat sarcoma viral oncogene (KRAS) (2). In Europe, panitumumab is indicated for metastatic colorectal cancer in the first-line setting with FOLFOX (5-FU, leucovorin, and oxaliplatin) as well as second line with FOLFIRI (5-FU, leucovorin, and irinotecan) following first-line fluoropyrimidine regimens that did not contain irinotecan (4).

The overall rate of severe adverse events (Grade 3 and more) with panitumumab is low. However, mild to moderate toxicities are common and include skin rash, diarrhea, and hypomagnesemia (4, 5). Side effects related to the central nervous system (CNS) beyond headache and dizziness, however, have not been reported with panitumumab so far. Accidental intra-arterial injection of substances due to incorrect central venous catheter location is rare. Commonly, injury distal to the injection site occurs, and multiple processes are believed to be involved in the pathogenesis of tissue damage (6). Here, we report a patient who developed acute encephalopathy and coma after accidental infusion of panitumumab into the left cardiac ventricle *via* a dislocated central venous line (Port-a-Cath©).

## Case Report

A 48-year-old woman was diagnosed with weakly differentiated (both KRAS and NRAS Exon 2/3/4 wild-type) rectosigmoidal cancer (cTx cN1 cM1 with disseminated pulmonal and hepatic metastases) in March 2016. She had received her first course of palliative FOLFOX chemotherapy consisting of folinic acid (200 mg/m^2^), 5-fluorouracil (200 mg/m^2^ as bolus and 400 mg/kg through a pump over 22 h), and oxaliplatin (85 mg/m^2^ over 2 h) plus panitumumab (6 mg/kg over 60 min). A central line (Port-a-Cath©) was implanted 1 week prior to the first cycle, and all treatments were well tolerated at this time. Her heart examination was unremarkable when she had an ischemic stroke 11 months earlier caused by spontaneous right internal carotid artery dissection. Back then, acute management included intravenous recombinant tissue-plasminogen activator (rt-PA), mechanical thrombectomy, and carotid artery stenting.

**Figure 1:**
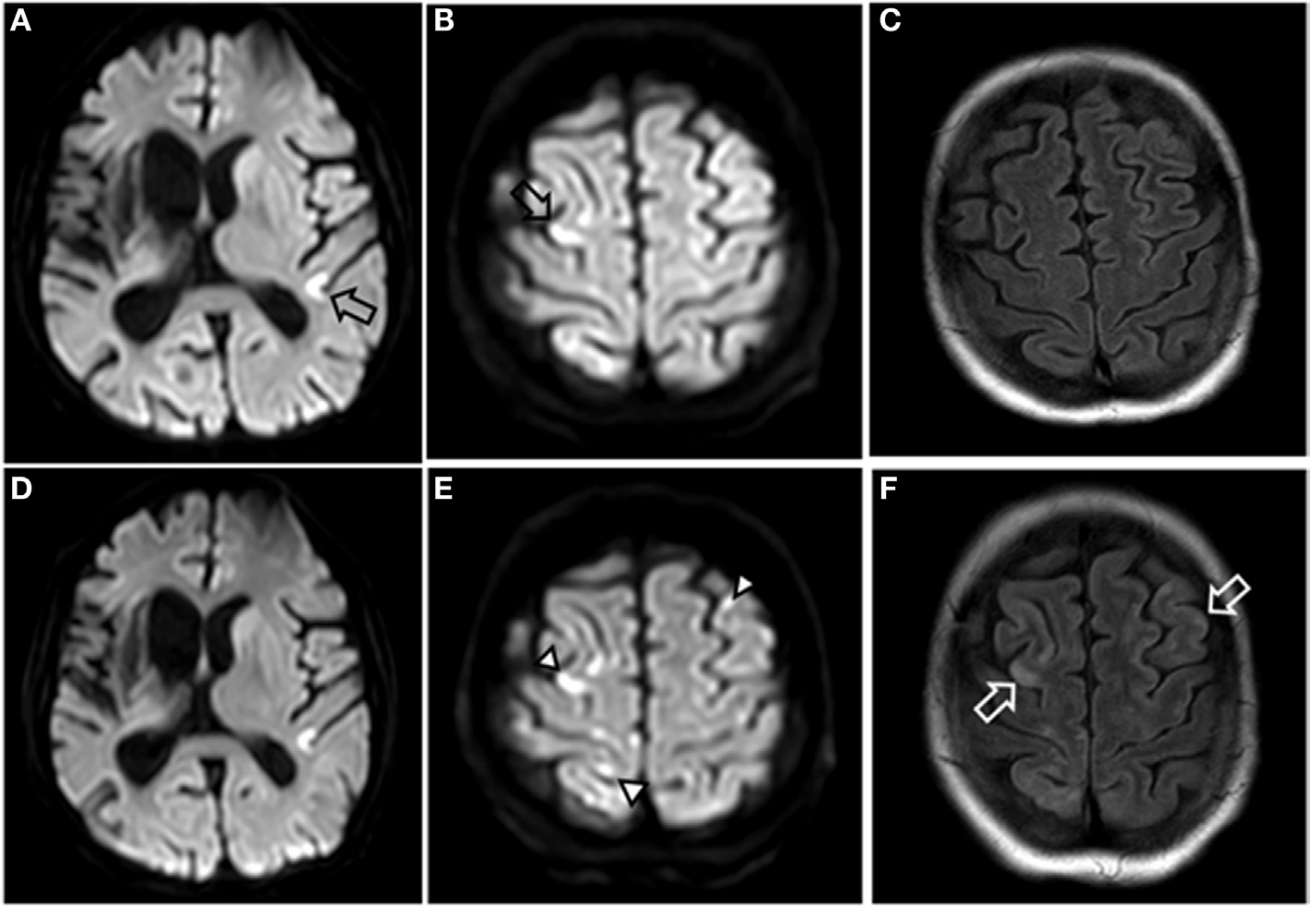
**MRI images of brain**. Diffusion-weighted imaging (DWI) 5 h after symptom onset showing multiple small hyperintensities in both hemispheres (open black arrows) **(A,B)**, and fluid-attenuated inversion recovery (FLAIR) showed no lesions **(C)**. Four days after onset, the multiple hyperintensities in DWI are better demarcated (closed black arrowheads) **(D,E)**, and FLAIR is showing multiple hyperintensities consistent with small areas of brain injury (open white arrows) **(F)**.

Three weeks later, she was admitted for the second chemotherapy cycle. The infusion line was flushed with sodium chloride solution before panitumumab administration (diluted in 100 ml sodium chloride). Shortly after completion of the panitumumab infusion using an infusion pump and a 0.2-μm in-line filter *via* the indwelling central venous catheter, she developed flexion of both arms and tremor in all extremities, and subsequent loss of consciousness. Upon neurologic examination she was comatose, had roving eyes, and bilateral Babinski signs. EEG did not show epileptiform activity. Brain MRI performed 5 h from symptom onset revealed multiple cortical hyperintensities on diffusion-weighted imaging (DWI). These were located in both hemispheres and preferentially in the frontal lobes (Figures 1A–C). No occlusion of major intracranial vessels was detected on time-of-flight angiography.

Pathological values in the lab exams included CRP 9.51 mg/dl (0.00–0.50), LDH 494 U/l (135–225), CK 531 U/l (26–140), cholesterol 477 mg/dl (150–220), LDL 398 mg/dl (50–150), serum iron 20 μg/dl (37–145), ferritin 178 ng/ml (23–110), and erythrocyte sedimentation 57 mm (0–20). Cerebrospinal fluid was obtained *via* lumbar puncture, and the only pathological value was a lactate of 5.1 mmol/l (1.1–2.4). On the second day, neuron-specific enolase (NSE) was 157.1 μg/l and increased to 513.5 μg/l (5.3–17.0) on day 5.

**Figure 2:**
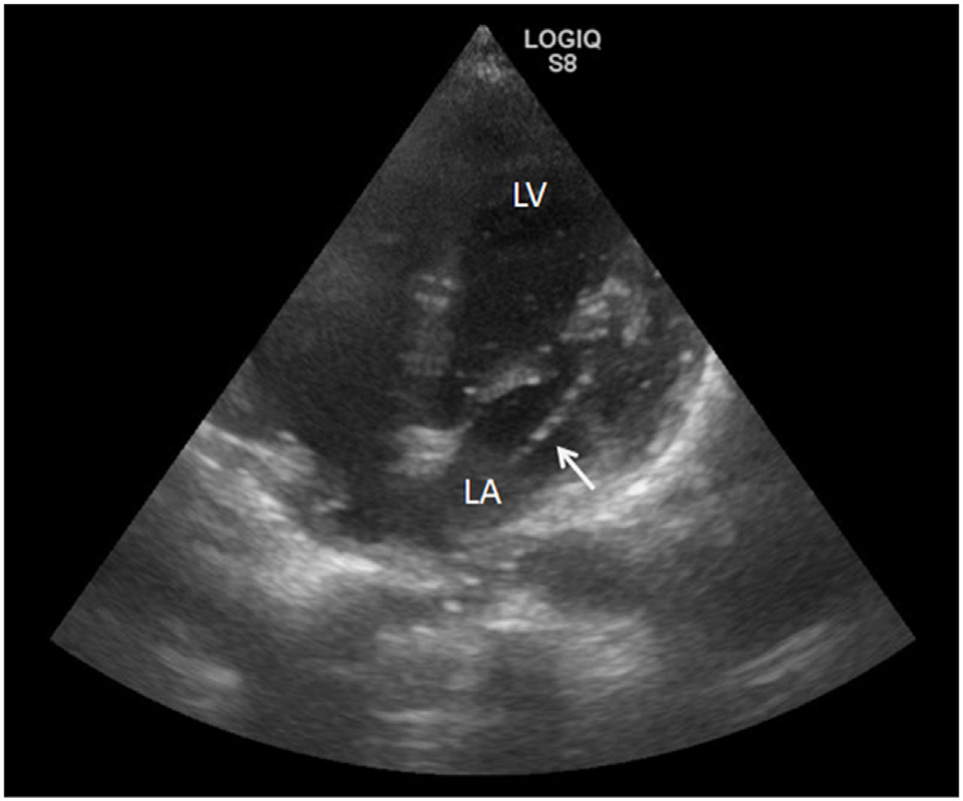
**Heart ultrasound showing the misplacement of a central venous access device (white arrow) *via* the interatrial septum, the left atrium (LA), and the mitral valve up to left ventricle (LV) during echo bubble test**.

We performed heart ultrasound 20 h from symptom onset and detected a dislocation of the indwelling central venous catheter tip, which had shifted through the interatrial septum to the left atrium, and *via* the mitral valve to the left ventricle (Figure 2). Transthoracal heart ultrasound was otherwise unremarkable.

The device was removed on the same day; the catheter tip was unremarkable on macroscopic examination. On day 3, she regained consciousness, and speech and swallowing impairment recovered over the next days. Brain MRI was performed on day 4 and showed fluid-attenuated inversion recovery (FLAIR) hyperintensities in both cerebral hemispheres (Figures 1D–F). She was transferred from the intensive care unit (ICU) to the oncological ward on day 5 with a residual spastic tetraparesis. There, pulmonary embolism and deep vein thrombosis (DVT) was diagnosed by CT pulmonary angiography and Doppler sonography, respectively.

## Discussion

Indwelling central venous access devices including the subcutaneous Port-a-Cath system are the mainstay of intravenous application for chemotherapy regimen. Major complications include thrombosis (1.6%), infections (1.6%), persistent pain and discomfort (1.2%), and dislocations (0.8%), necessitating premature removal in up to 5% (7). Moreover, thrombi may form around the catheter and cause ischemic stroke in the presence of a patent foramen ovale (PFO) (8). With regard to the etiology of the acute encephalopathy, we could rule out catheter thrombosis and sequential embolism to the brain, as no thrombotic material was detected in heart ultrasound and upon evacuation of the catheter. Increased CRP may raise suspicion for septic embolism caused by vegetations. However, blood cultures were negative for bacterial growth, and CSF examination revealed only high lactate. Together with increased NSE serum levels, this finding was consistent with brain injury. With regard to cerebral infarction, it is very unlikely that thrombi, which detach from DVT and relocate *via* the PFO, can cause such widespread cortical lesions. DVT may be a consequence of cancer *per se* or consequence of hyperviscosity, as discussed later (9). Pulmonary hypertension caused by embolism may have supported dislocation of the device. Thus, central venous devices in patients with DVT should undergo verification of its location. In addition, regular heart ultrasound for reconfirmation of correct placement may reduce the chance of dislocation and accidental administration of substances.

Acute neurovascular toxicity and posterior reversible encephalopathy syndrome (PRES) are the most probable underlying causes of brain injury in our patient. Panitumumab binds with high affinity to the extracellular domain of EGFR in both tumor and normal tissue cells. While many neurons of the CNS constitutively express the EGFR, glial and endothelial cells demonstrate induced receptor expression following acute injury or chronic neurodegeneration (10). Toxic encephalopathy is supported by the presumed panitumumab concentration in the cerebrovascular system after intracardiac administration, whereas a diluting effect can be assumed when infused *via* the venous route. Indeed, there are several mechanisms of action identified by *in vitro* studies, which could induce vascular dysfunction and subsequent brain damage. These include (1) downregulation of EGFR through receptor internalization, (2) induction of apoptosis *via* inhibition of EGFR signaling pathways, (3) induction of cell cycle arrest, (4) induction of autophagy, and (5) inhibition of angiogenesis (11). No information is available on the ability of panitumumab to cross blood–brain barrier, whereas intracranially grown GBM xenografts responded to systemically administered cetuximab treatments (12). An occlusion of microvessels due to undiluted protein particles needs to be taken into account as panitumumab can form protein particles. The usage of a protein filter should eliminate this option. Microvascular thrombosis may also be caused by increased viscosity, as seen occasionally with intravenous immunoglobulin (IVIG). A majority of strokes caused by IVIG treatment occur during or within 24 h of the infusion (13). In the case of encephalopathy caused by tocilizumab, a monoclonal antibody against interleukin-6-receptor, brain biopsy revealed multifocal cerebral thrombotic microangiopathy (14). The quick resolution of symptoms as well as lack of further immune-mediated complications and multi-organ involvement renders the latter condition unlikely.

Another option for the symptoms displayed by this patient is PRES. Of note, PRES is increasingly recognized as a complication of patients with cancer (15–17). In an US American cohort, more than half of the patients (55%) had received chemotherapy or targeted therapy within the months before PRES developed (16). This clinico-radiological entity is characterized by acute encephalopathy, seizures, and additional neurologic symptoms and neuroimaging, demonstrating lesions with posterior and white matter predominance. This contrasts our case where EEG was normal and brain lesions were mostly frontal and cortical. PRES has been reported with bevacizumab, a humanized antibody that targets vascular endothelial growth factor (VEGF), with a minimum incidence of 0.01% (16, 18). Moreover, PRES as well as seizures have been observed with systemic cetuximab therapy (19, 20). So far, no cases of panitumumab-associated PRES are known, and our patient was normotensive. Notably, a retrospective study of 96 patients with PRES revealed that those who received recent chemotherapy or immunosuppressive medications had significantly lower mean arterial pressure than those with PRES of other etiologies (21). Electrolyte disorders and particularly hypomagnesemia is a common side effect of panitumumab treatment (22). While hypomagnesemia can cause CNS dysfunction *per se*, it may also be relevant for the pathogenesis of PRES (23). While magnesium levels were normal in our patient, serum levels are not a reliable means to determine total magnesium depletion (24).

Despite the fact that the clinical symptoms developed in close relation to the antibody infusion and prior to the second FOLFOX cycle, we cannot rule out the impact of comorbidity and previous medication including chemotherapy. We conclude that acute encephalopathy may be a hitherto unrecognized complication of treatment with panitumumab and should be taken into consideration in patients developing CNS symptoms undergoing this therapy. Direct interaction of the antibody with neuronal structures or indirect pathomechanisms could be envisioned. Cerebral ischemia caused by paradoxical embolism, hyperviscosity, or other mechanism of action, however, cannot be completely ruled out.

## Author Contributions

SP: drafting/revising the manuscript, accepts responsibility for conduct of research and final approval; GP, GG, CR, KS, and RG: revising manuscript and acquisition of data, accepts responsibility for conduct of research and final approval; JS: drafting/revising the manuscript, acquisition of data, and accepts responsibility for conduct of research and final approval.

## Conflict of Interest Statement

RG reported receiving honoraria from Bristol-Myers-Squibb, Cephalon, Amgen, Eisai, Mundipharma, Merck, Janssen-Cilag, Genentech, Novartis, AstraZeneca, Boehringer Ingelheim, Pfizer, Roche, and Sanofi Aventis; research funding from Cephalon, Celgene, Amgen, Mundipharma, Genentech, Pfizer, GSK, and Ratiopharm. Also, RG has been a consultant for Bristol-Myers-Squibb, Cephalon, and Celgene. The other authors declare that they have no competing interests.

## Supplementary Material

Video S1 **The transthoracal heart ultrasound during the bubble test detects the distal part of the misplaced central venous access device and air bubbles in left ventricle**. The catheter is floating in the LA, and the tip is protruding through mitral valve into the LV with numerous gaseous bubbles originating from it. Duration: 30 s. Abbreviations: RA, right atrium; LA, left atrium; LV, left ventricle.
